# Circulating Tumor DNA Analysis in *ERBB2*-Amplified Colorectal Cancer: Biomarker Analysis of the MyPathway Trial

**DOI:** 10.1158/1078-0432.CCR-24-2763

**Published:** 2025-07-15

**Authors:** F. Meric-Bernstam, K. Raghav, C. J. Sweeney, C. Swanton, D. R. Spigel, R. Bose, H. A. Burris, J. Hainsworth, C. F. Friedman, C. R. Espenschied, J. M. Grindheim, J. Malato, K. Schulze, R. Price, R. Kurzrock

**Affiliations:** 1 Department of Investigational Cancer Therapeutics, The University of Texas MD Anderson Cancer Center, Houston, TX, USA; 2 Department of Gastrointestinal Medical Oncology, The University of Texas MD Anderson Cancer Center, Houston, TX, USA; 3 South Australian Immunogenomics Cancer Institute, University of Adelaide, Adelaide, Australia; 4 Francis Crick Institute, London, UK; 5 Sarah Cannon Research Institute, Nashville, TN, USA; 6 Washington University School of Medicine, St Louis, MO, USA; 7 Department of Medicine, Memorial Sloan Kettering Cancer Center, New York, NY, USA; 8 Department of Medicine, Weill Cornell Medical College, New York, NY, USA; 9 Global Scientific Affairs, Guardant Health, Redwood City, CA, USA; 10 Genentech, Inc, South San Francisco, CA, USA; 11 Medical College of Wisconsin Cancer Center and Linda T. and John A. Mellowes Center for Genomic Sciences and Precision Medicine, Milwaukee, WI, USA

## Abstract

**Background:**

Combination of two HER2-directed antibodies, pertuzumab and trastuzumab (P+T), has antitumor activity in HER2-positive colorectal cancer (CRC). Although liquid biopsies are increasingly being used in clinical oncology, the association between tumor and circulating tumor DNA (ctDNA) *ERBB2* status and ctDNA monitoring for early response and resistance are unknown.

**Patients and Methods:**

Eighty-five patients with *ERBB2*-amplified and/or overexpressed CRC were treated with P+T in the MyPathway trial; 42 had ctDNA testing at Cycle 1 Day 1 (C1D1), and 38 had longitudinal plasma tested for ctDNA. We analyzed the ctDNA vs tissue *ERBB2* concordance, genomic co-alterations, and ctDNA dynamics and association with response.

**Results:**

Forty-one (98%) of 42 patients had genomic alterations detected in ctDNA at C1D1, and 29 (69%) had *ERBB2* amplification in ctDNA. There was a strong correlation between *ERBB2* copy number on next-generation sequencing in tissue and C1D1 *ERBB2* ctDNA copy number. Thirty-seven percent achieved a molecular response by C3D1 on P+T, which was associated with prolonged progression-free survival and overall survival (OS). *CDKN2A* and *KRAS* mutations were associated with shorter OS, and a trend was seen with *PIK3CA* mutations. Several emerging co-alterations were identified in ctDNA at progression, including in the MAPK and PI3K pathways and other tyrosine receptor kinases.

**Conclusions:**

ctDNA can detect *ERBB2* amplification in many, but not all, patients with *ERBB2* amplification detected in tumor samples. ctDNA molecular response was associated with better survival, and ctDNA co-alterations may offer insights into mechanisms of intrinsic and acquired resistance.

ClinicalTrials.gov, NCT02091141

## Introduction

HER2*/ERBB2* amplification and/or overexpression is observed in 2 to 3% of all solid tumors and in 3 to 5.8% of colorectal cancers (CRCs).^1–4^ HER2 has emerged as a validated target in metastatic CRC with numerous recent U.S. Food and Drug Administration approvals including the combination of tucatinib and trastuzumab, trastuzumab deruxtecan and with the NCCN Clinical Practice Guidelines in Oncology (NCCN Guidelines®) inclusion of pertuzumab in combination with trastuzumab (P+T) and lapatinib in combination with trastuzumab.^5–8^ The MyPathway trial (NCT02091141) demonstrated an objective response rate (ORR) of 33% in patients with HER2+, *KRAS* wild-type CRC. The American Society of Clinical Oncology TAPUR trial also confirmed the antitumor activity of P+T, demonstrating an ORR of 25% in *ERBB2*-amplified CRC. Although HER2 is clearly an actionable target in CRC, there have been few studies into the mechanisms of intrinsic and acquired resistance to HER2-targeted therapies in CRC.^8–11^

Assessment of genomic alterations in circulating tumor DNA (ctDNA) in plasma is now an important tool in precision oncology.^12,13^ There are strong data demonstrating that these alterations can be used to identify actionable targets, including mutations, copy number alterations, and some select fusions, and to inform treatment decisions.^14,15^ In addition, longitudinal sample collection is being used for pharmacodynamic monitoring of disease burden on therapy as well as an approach to identify emerging mutations that may be associated with acquired resistance in gastrointestinal tract tumors.^16–19^

In this study, we evaluated the role of liquid biopsies in a cohort of patients with *ERBB2*-amplified and/or overexpressed CRC treated with plasma available for ctDNA analysis in the MyPathway trial. The goals of this study were to: (1) correlate pretreatment ctDNA findings to tumor tissue assay results, (2) correlate serial changes in ctDNA with treatment response (as measured by standard Response Evaluation Criteria in Solid Tumors [RECIST] version 1.1 criteria) and survival, (3) evaluate the influence of coexisting genomic alterations on treatment response, and (4) recognize emerging alterations at the time of cancer progression.

## Materials and Methods

### Study Design and Participants

MyPathway (NCT02091141) is an open-label, non-randomized, multicenter, multiple-basket, phase 2a study including a CRC cohort (full protocol online). Patients in the CRC cohort of the HER2 basket described here had tumors with *ERBB2* amplification and/or HER2 overexpression. Intravenous pertuzumab (840-mg loading dose, then 420 mg every 3 weeks [q3w]) and trastuzumab (8-mg/kg loading dose, then 6 mg/kg q3w)^20,21^ were administered until progressive disease or unacceptable toxicity. Tumor response was investigator-assessed per RECIST v1.1 at baseline and every two treatment cycles for the first 24 weeks, then every four cycles thereafter.^22^

MyPathway was performed per the International Conference on Harmonisation guideline for Good Clinical Practice and the Declaration of Helsinki. The institutional review board or ethics committee at each study site approved the protocol. Patients provided written informed consent prior to enrollment. The protocol was modified to incorporate longitudinal plasma collection on participants in the latter part of the study. In addition, prior to that amendment, patients enrolled at MD Anderson Cancer Center had longitudinal plasma collections under a separate protocol (NCT01772771) approved by the MD Anderson Institutional Review Board.

Enrollment in the MyPathway *ERBB2* amplification/HER2 overexpression cohort was based on HER2-altered archival or fresh tumor tissue samples, as assessed by Clinical Laboratory Improvement Amendments–certified laboratories. HER2 overexpression was identified by 3+ staining by immunohistochemistry (IHC). *ERBB2* amplification was based on fluorescence/chromogenic in situ hybridization (*ERBB2* amplification based on *ERBB2*/*CEP17* ratio >2.0 or *ERBB2* copy number >6.0), next-generation sequencing (NGS; *ERBB2* amplification based on any copy number gain), and/or polymerase chain reaction or Sanger sequencing (*ERBB2* amplification or mutations). Testing platforms, HER2 status, and other molecular alterations (eg, *KRAS* mutations) identified from local testing were reported at enrollment.

In some patients with available archival tissue, local HER2, and co-alteration testing data were supplemented with central FoundationOne NGS testing (Foundation Medicine, Cambridge, MA, USA).^23^ Additionally, in some patients, *ERBB2* amplification and overexpression were assessed post enrollment using a single slide-based HER2 gene–protein assay incorporating in situ hybridization (ISH) and IHC (Ventana Medical Systems, Tucson, AZ, USA).^24^ In cases of discordant local versus central results for *ERBB2* amplification, overexpression, or mutation status, central results took precedence.

### ctDNA Substudy in Patients With ERBB2-Amplified or Overexpressed CRC

The collection of blood for ctDNA analysis was an optional part of the MyPathway study until protocol version 3 (2017). In consenting patients, plasma collections were performed prior to treatment (Cycle 1 Day 1 [C1D1]), during treatment (C3D1), and at the time of tumor progression. Plasma samples were processed for ctDNA sequencing at Guardant Health (Redwood City, CA, USA) using Guardant360 (G360), as previously described.^25,26^

C1D1 ctDNA results were compared with the tissue testing results performed on archival tissue. ctDNA changes during treatment were correlated with treatment efficacy results, as measured by standard RECIST criteria. Co-existent molecular alterations detected at C1D1 by ctDNA were compared with those detected by tissue testing. The influence of co-mutations on the efficacy of P+T treatment and ctDNA-detected co-mutations emerging at tumor progression were assessed.

### Statistical Analysis

#### Molecular Response

Fold-change (FC) molecular response (MR) was calculated as follows. Mean variant allele frequency (VAF) was calculated as the mean ctDNA percentage for single nucleotide variants (SNVs), indels, and fusions. If data was run but no variants in SNVs, indels, or fusions were detected, mean VAF was set to 0. MR (log2 fold change) in mean VAF between two time points was calculated as log2( (end VAF + pseudocount) / (start VAF + pseudocount)), where the pseudocount was set to 0.1, as it was the smallest non-0 mean VAF in the dataset. A threshold of two-FC was used to classify MR as increase, no change, or decrease. As an alternate strategy we also assessed MR using the Guardant360 Response algorithm, which is based on the method defined previously by Zhang et al.^27^ We calculated the ratio of the on-treatment VAF to pre-treatment VAF, defining MR as a >50% decrease in VAF.^28,29^

## Results

Three hundred forty-six patients were enrolled in the HER2 cohort of MyPathway with protocol-stipulated HER2 molecular alterations [reference MyPathway Protocol V4, V5] and 85 were enrolled with *ERBB2*-amplified and/or -overexpressing CRC based on local tumor analysis. The protocol was modified to collect longitudinal ctDNA samples in the latter part of the protocol. The consort diagram is shown in Supplementary Figure 1A. This study is focused on 42 patients who had HER2-amplified and/or HER2-overexpressing CRC, treated with P/T, and had plasma analysis for ctDNA at baseline. Of these patients, 37 also had samples at C3D1 and 13 had end-of-treatment (EOT) samples. Samples were analyzed as progression samples if they occurred within 4 weeks of disease progression (PD). Mean time and number of cycles between baseline and EOT sample were 5.7m ± 9.7 (x=9.2 cy ± 13.8). Supplementary Figure 1B demonstrates patient and sample characteristics.

### Detection of Genomic Alterations and Estimation of Disease Burden in ctDNA

**Figure 1:**
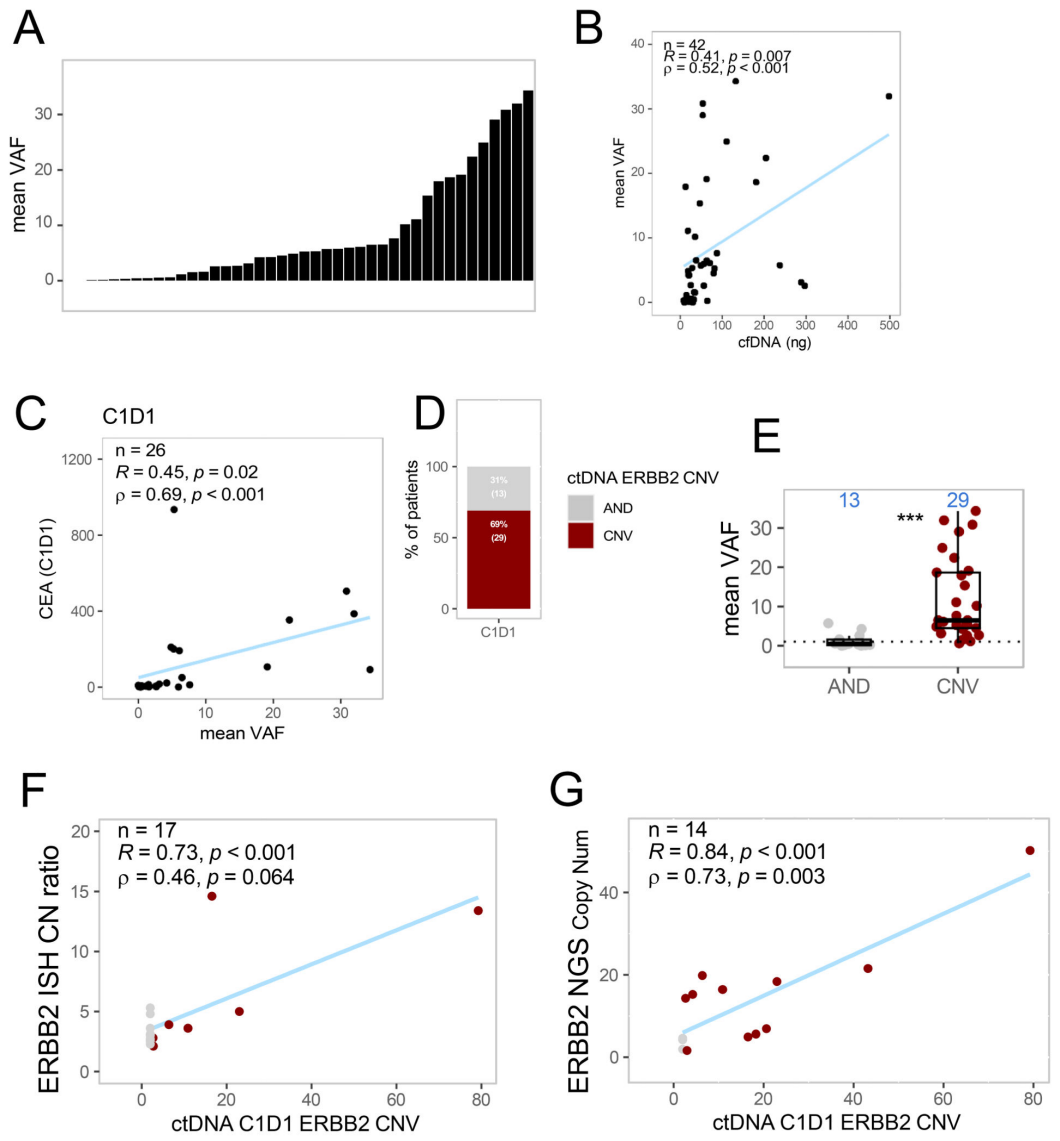
Baseline characteristics of ctDNA detected in plasma. (A) Range of mean VAF at baseline across the plasma cohort. (B) Lower input DNA is correlated with low mean VAF. (C) correlation of ctDNA with CEA. (D) Detection of *ERBB2* amplification in ctDNA. (E) *ERBB2* amplification status vs mean VAF. *ERBB2* copy number in ctDNA correlates with tissue assessments by either ISH (F) and NGS panel (G). AND, amplification not detected; CEA, carcinoembryonic antigen; CNV, copy number variant; ctDNA, circulating tumor DNA; ISH, in situ hybridization; NGS, next-generation sequencing; VAF, variant allele frequency.

Forty-one of 42 patients (98%) had any genomic alterations detected on ctDNA at C1D1. Figure 1A demonstrates detection of genomic alterations in ctDNA, presented by mean VAF. Patients had an average of 2.9 mL of plasma available for analysis (range, 1.8-5 mL). There was no correlation of ctDNA extracted and the plasma input volume within this range. However, the mean VAF significantly correlated with the ctDNA input volume as shown in Figure 1B (*R*=0.41, p=0.007), thus we hypothesized that both baseline ctDNA volume and mean VAF may be surrogates of disease burden. To assess whether tumor burden contributed to ctDNA detection, we assessed a clinical marker of tumor burden, carcinoembryonic antigen (CEA) levels that were obtained locally at treatment initiation.^30^ CEA levels were available in 26 patients and were significantly correlated with baseline mean VAF (*R*=0.45, p=0.02), suggesting a relationship between mean VAF and disease burden (Figure 1C).

**Figure 2:**
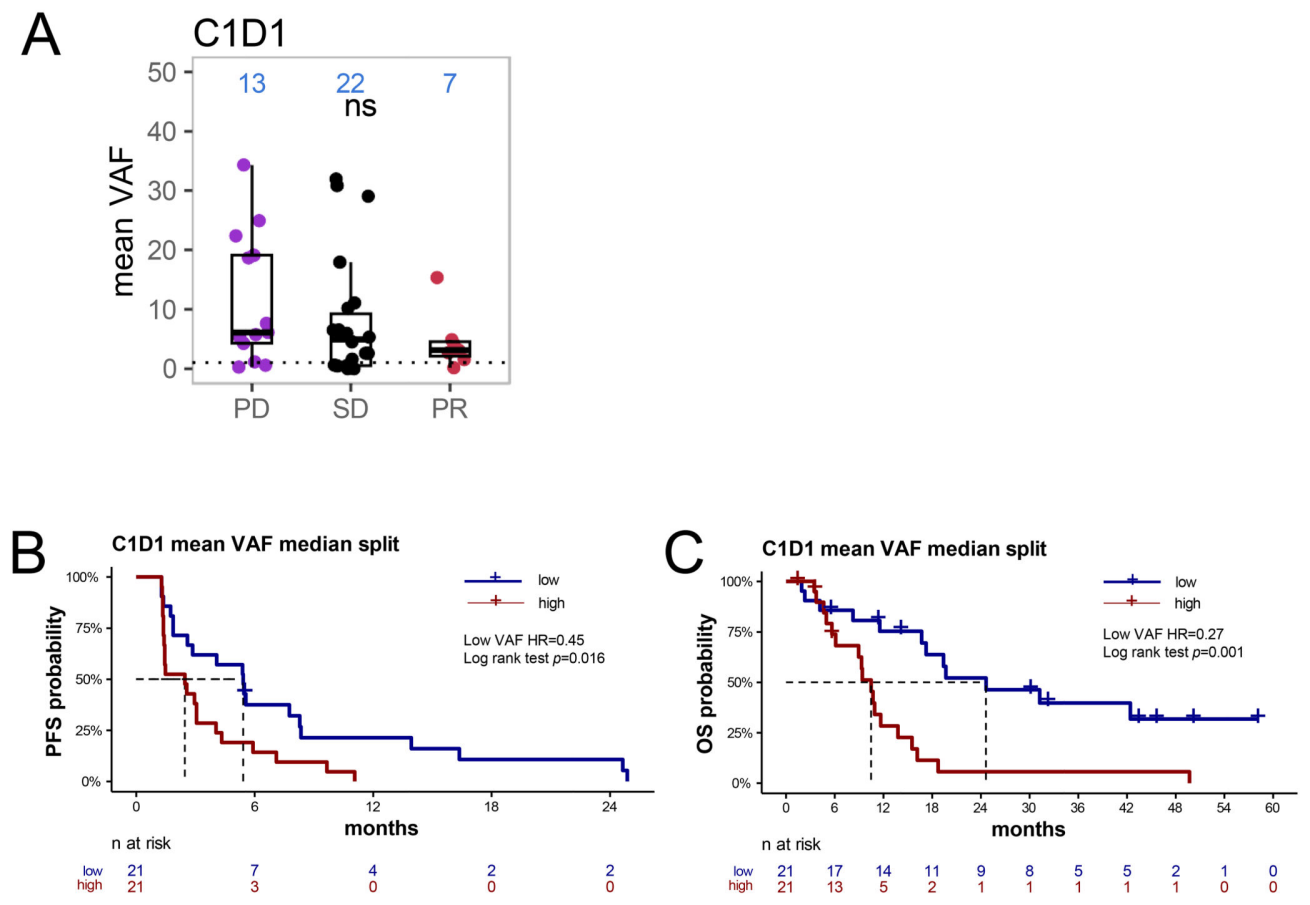
Relationship of baseline ctDNA levels to outcome. (A) Mean VAF by clinical response. Kaplan-Meier curves showing PFS (B) and OS (C) for patients grouped into mean VAF high vs low (median split). C1D1, Cycle 1 Day 1; ctDNA, circulating tumor DNA; OS, overall survival; PD, disease progression; PFS, progression-free survival; PR, partial response; SD, stable disease; VAF, variant allele frequency.

We next assessed the association of ctDNA VAF with efficacy outcomes. There was no statistically significant difference between the mean VAF among patients divided by best overall response (BOR) (PD/stable disease [SD]/partial response [PR]) (Figure 2A). However, when mean VAF at baseline was split by median, patients who had lower mean VAF, had significantly longer progression-free survival (PFS) (HR, 0.45; 95% confidence interval [CI], 0.23-0.88; p= 0.016; Figure 2B) and overall survival (OS) (HR, 0.27; 95% CI, 0.12-2.58; p< 0.001; Figure 2C).

**Figure 3:**
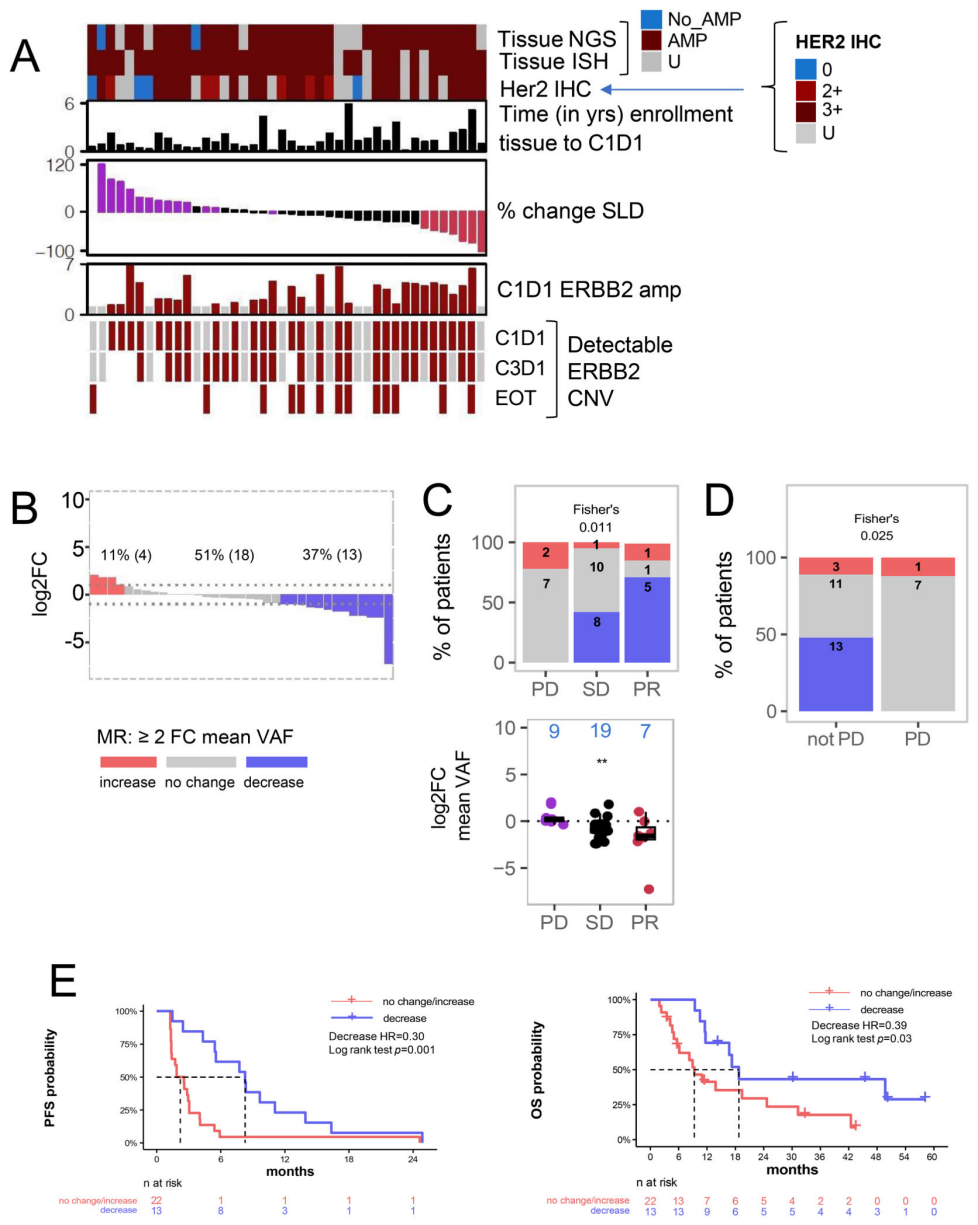
Longitudinal detection of *ERBB2* amplification and molecular response assessments across the cohort. (A) Overview of Her2 detection across the plasma cohort in relation to changes in tumor size and various enrollment assessments. (B) ctDNA dynamics as assessed by molecular response using a fold-change method. (C) Relationship of molecular response to best overall response in both proportion of patients (upper) or by change in mean VAF (lower). (D) Molecular response proportions when PD was recorded within 4 weeks of C3D1. (E) Kaplan Meier curves showing PFS and OS according to molecular response increase or decrease. AMP, amplification; CNV, copy number variant; ctDNA, circulating tumor DNA; EOT, end of treatment; FC, fold-change; IHC, immunohistochemistry; ISH, in situ hybridization; NGS, next-generation sequencing; OS, overall survival; PD, disease progression; PFS, progression-free survival; PR, partial response; SD, stable disease; U, Untested.

We next assessed the detection of *ERBB2* amplification in ctDNA. At C1D1, we were able to detect *ERBB2* amplification in ctDNA in 29 of the 42 patients (69%) (Figure 1D). Patients who had *ERBB2* amplification detected on ctDNA had significantly higher mean VAF compared with those who did not have *ERBB2* variation detected (Figure 1E), suggesting that lack of detection is at least in part a result of low tumor shedding. Three of the 13 patients without *ERBB2* amplification detected at baseline had other copy number variations called, suggesting at least these three were true negatives (Supplementary Table 2). We observed a relationship between *ERBB2* status and NGS and ISH results, as there was a significant correlation between ctDNA C1D1 *ERBB2* copy number and *ERBB2* ISH copy number (*R*=0.73, p<0.001), and *ERBB2* copy number on NGS in tissue (*R*=0.84, p<0.001). There was no association of detection of *ERBB2* amplification on C1D1 ctDNA with oncological outcomes. Figure 3A demonstrates detection of *ERBB2* amplification on ctDNA analysis on serial samples. Of the 29 patients who had ctDNA *ERBB2* amplification at C1D1, 3 did not have amplification detectable at C3D1, including 2 patients with PR and one with SD. Two patients with BOR of SD became *ERBB2*-amplification detectable. All 13 patients with EOT samples had *ERBB2* amplification detectable on ctDNA, including two patients without detectable amplification at C1D1.

### Molecular Response and Outcomes

Next, we looked at the dynamics of the ctDNA molecular profile, relationship with response, and survival outcomes using both fold change and % change as described in Methods.

When using a 2-fold reduction in mean VAF between baseline and C3D1 (Figure 3B) as a MR 13patients (37%) had a 2-fold decrease in mean VAF, four (11%) had a 2-fold increase, and 18 (51%) had no change (Figure 3B). This response profile was correlated with BOR with all patients with a reduction in ctDNA having SD or PR, and with 5/7 PR patients also having an MR (Figure 3C). When we defined C3D1 samples as PD samples if progression occurred within 4 weeks of C3D1, no patients with PD within 4 weeks of C3D1 had an MR, compared to 48% of patients without PD at C3D1 (Figure 3D).

Patients who had a ctDNA MR (2-fold reduction in mean VAF), from C1D1 to C3D1 had a longer PFS (HR, 0.30; 95% CI, 0.14-0.63; p=0.001) and longer OS (HR, 0.39; 95% CI, 0.16-0.94; p=0.028, Figure 3E).

We also assessed ctDNA MR based on criteria described by Zhang et al.,^27^ with molecular responders defined as those with at least a 50% decrease in mean VAF (Supplementary Figure 2). Comparable proportion of patients demonstrated a decrease in their ctDNA with treatment by these MR criteria. Twelve of 33 patients (38%) were molecular responders, 14 (44%) were molecular non-responders, and six (19%) were ctDNA low. With this method, ctDNA low indicates that ctDNA levels were too low to accurately quantify the % change and patients with low ctDNA levels typically have better outcomes than those with higher ctDNA levels,^28^ which was observed in this cohort for both PFS and OS. Patients who had a MR from C1D1 to C3D1 had a longer PFS as compared to patients without MR (HR, 0.05; 95% CI, 0.01-0.30; p<0.001) but no OS association was observed.

### Detection of Other Genomic Alterations on ctDNA

**Figure 4:**
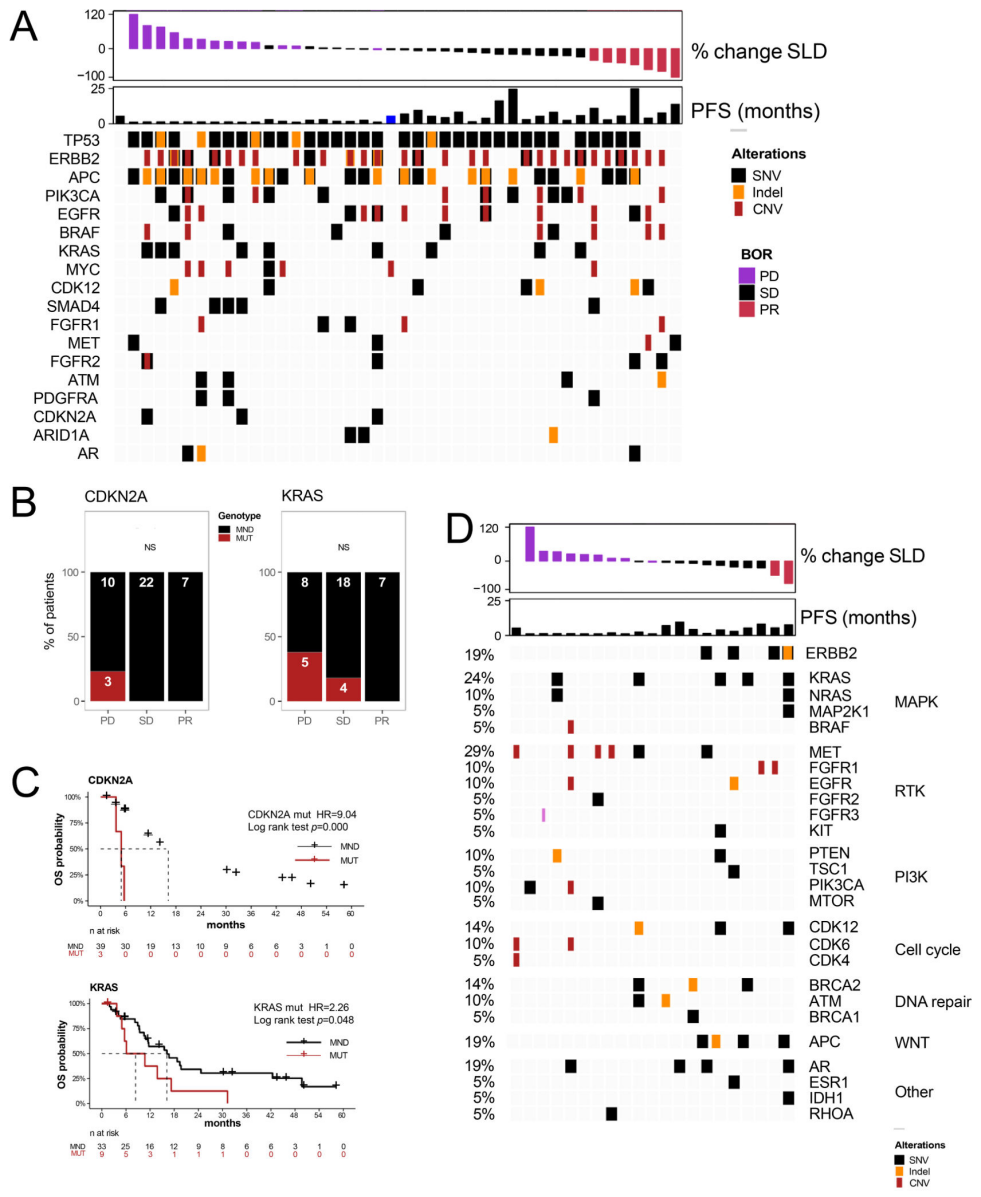
Overview of clinical factors and molecular alterations detected from ctDNA across the plasma cohort. (A) Baseline oncoprint showing top altered genes by tumor response. (B) CDKN2A and KRAS alterations by response category highlighting OS of mut vs WT CDKN2A and KRAS (C). (D) Acquired mutations detected at PD but not seen at C1D1 or in baseline tissue. BOR, best overall response; C1D1, Cycle 1 Day 1; CNV, copy number variant; ctDNA, circulating tumor DNA; FC, fold-change; MR, molecular response; NA, not available; NS, no significant comparisons; OS, overall survival; PD, disease progression; PFS, progression-free survival; PR, partial response; SD, stable disease; SLD, sum of longest diameters; SNV, single nucleotide variant; VAF, variant allele frequency.

Next, we evaluated the C1D1 ctDNA genomic profile to determine whether there were other oncogenic drivers detected and whether the C1D1 ctDNA co-alterations were associated with efficacy outcomes. As shown in Figure 4A, the most common other alterations were in *TP53* (83%), *APC* (64%), *PIK3CA* (33%), *EGFR* (31%), *BRAF* (45%), and *KRAS* (21%). Patients had additional alterations in potential therapeutic targets including mutations or amplification of *FGFR1, FGFR2*, and *MET* alterations, and mutations in *ATM*. However, few were known oncogenic driver mutations; all are noted in Supplementary Table 1.

Of the seven patients with a BOR of PR, six had *ERBB2* amplification detectable on ctDNA, one with a concomitant *ERBB2* SNV G776V, an activating mutation. Only one of the patients with a response had a *PIK3CA* alteration, and none had *KRAS* or *CDKN2A* mutations.

We then evaluated the association of genomic co-alterations at C1D1 with OS. Although there were only three patients with *CDKN2A* mutations, all three progressed (Figure 4B) and they had a significantly shorter OS (HR, 9.04; 95% CI, 2.1-38.3; p<0.001; Figure 4C). In tissue testing, we had previously demonstrated that *KRAS* mutations were associated with worsened outcomes.^10,31^ In this ctDNA substudy, 9/42 patients had *KRAS* mutations detected in C1D1 ctDNA (Figure 4B) and patients with *KRAS* mutations had significantly worse OS (HR, 2.3; 95% CI, 1.0-5.2; p=0.048; Figure 4C). Seven patients had *PIK3CA* mutations and five had *PIK3CA* amplifications, with an additional two patients with both a *PIK3CA* mutation and amplification detected on ctDNA. Patients with *PIK3CA* alterations also demonstrated a trend toward worsened OS (HR, 2.1; 95% CI, 1.0-4.5; p=0.06; Not shown).

### Acquired Alterations on HER2-Targeted Therapy

Next, we assessed the changes in ctDNA in 21 patients who had both C1D1 and PD samples, including 8 patients who had C3D1 samples as PD samples (Figure 4D). We defined “acquired” as alterations that were detected in PD samples but were absent from NGS analysis of the archival tumor tissue and not detected in C1D1 ctDNA. These alterations are listed in Supplementary Table 2. Notably, four of 21 patients (19%) had *ERBB2* SNVs detected: two had *ERBB2* SNVs not known to be oncogenic (HER2 V505L and R487W), one had a *ERBB2* mutation that is known to be oncogenic (HER2 S310F), and another had two *ERBB2 S*NVs (HER2 N530T and HER2 V356G) and a lower allelic frequency *HER2* indel A775_G776insYVMA at an oncogenic hotspot. Six of 21 patients (28.6%) had alterations of the MAP kinase pathway emerge at PD; five patients had activating *KRAS* mutations emerge at PD, and two of these patients had additional convergent MAPK alterations.

There were also several acquired alterations in other receptor tyrosine kinases. Notably 6 of the 21 patients (29%) had *MET* alterations. Four patients had *MET* amplifications emerge at PD; three of these patients had progressive disease as their best response. Two patients had *MET* SNVs, one with a mutation that is known to be activating (MET Y1003F).^32,33^ In addition, four patients had alterations in *FGFR1/*2/3, including two *FGFR1* amplifications, one *FGFR3* fusion, and one *FGFR2* SNV (K367M VUS). Furthermore, six patients (28.6%) had emergence of PI3K pathway alterations, including activating *PIK3CA* mutation (M1043I), *PIK3CA* amplification, and inactivating *PTEN* mutation (N94fs)

## Discussion

HER2 has emerged as an important target for the treatment of HER2-positive metastatic CRC. In the MyPathway trial, we previously demonstrated the antitumor activity of P+T, leading to incorporation of this regimen into both the ESMO Clinical Practice Guidelines and the NCCN Guidelines®.^8, 34^ Here we demonstrate that the majority of patients with *ERBB2*-amplified CRC have *ERBB2* amplification on ctDNA, but that ctDNA *ERBB2* detection is at least in part associated with tumor burden. Lower VAF in ctDNA and ctDNA response to treatment were both associated with prolonged PFS and OS by at least one method.

There is growing interest in using ctDNA in the management of CRC, both for assessment of minimal residual disease and detection of actionable alterations in the tumor.^35–37^ Notably, when SCRUM-Japan GI-Screen, a ctDNA based study was compared with GOZILA, a tissue sequencing based study, it was apparent that ctDNA-based screening significantly shortened screening duration and increased trial enrollment.^15^

Although there is increasing utilization of ctDNA for precision oncology, there are still few studies systematically comparing the overall agreement of ctDNA findings with those of tumor tissue.^12,38^ It has also been especially unclear whether ctDNA can effectively detect copy number changes.^39^ Here we demonstrate that *ERBB2* amplification was detectable in 69% of patients with *ERBB2*-amplified CRC. In our study, the copy number in ctDNA strongly correlated with the copy number on tissue testing. However, detection of *ERBB2* on liquid biopsy was at least in part dependent on tumor burden. The tissue sample analysis was based on archival tissue and not a fresh biopsy, thus discordance in ctDNA status may have been due, in part, to genomic evolution. Interestingly, the ctDNA sensitivity in our study was substantially lower than that observed in the HERACLES trial, where 47 of 48 samples (97.9%) from 29 patients with HER2-positive CRC had *ERBB2* amplification detectable on the same Guardant360 platform.^40^ Furthermore, the adjusted copy number of 25.82 copies predicted who would benefit from trastuzumab in combination with lapatinib. Guardant360 has evolved over time, as is typical with genomic assays, and this may partially explain the differences in detection rates. Additionally, the eligibility criteria for HERACLES required HER2 IHC of 3+ in at least 50% of cells or HER2 IHC 2+ with HER2:CEP17 ratio greater than two in at least 50% of cells while My Pathway simply required HER2 amplification or overexpression. The HERACLES criteria likely selected for higher amplification level which may have also contributed to the higher detection rate by ctDNA in that study.

As all patients in this series had ctDNA amplification in tumor testing, we did not assess the positive predictive value of ctDNA testing. However, Nakamura et al reported clinical activity with P+T in both patients with CRC with *ERBB2* amplification on tissue testing and those treated based on ctDNA amplification on ctDNA, suggesting that *ERBB2* amplification on ctDNA is actionable.^41^ Thus taken together with our data, *ERBB2* amplification on ctDNA is actionable, but lack of *ERBB2* amplification on ctDNA does not rule out *ERBB2* amplification on tumor analysis.

There has been great interest in longitudinal monitoring for early assessment of response. Although our cohort is small, it has the strength that it included patients with response as well as progression on study. We demonstrated MRs with P+T treatment using two different MR metrics. MR with both criteria was associated with a longer PFS. The Guardant360 Response algorithm, based on Zhang et al,^27^ also identified patients with low ctDNA levels who had numerically better PFS and OS, which did not reach statistical significance due to small numbers. Further study into molecular response monitoring is needed to confirm whether it can help determine patients who are responding early and whether it may allow for change in treatment or combination of therapy in those who are not responding.

Liquid biopsy testing is becoming increasingly sophisticated, allowing for testing of larger panels and even whole exome sequencing, to facilitate research and patient care. The panel used in this study, Guardant360, included 74 genes, allowing for assessment of multiple other actionable alterations. We thus assessed co-alterations in the baseline samples to determine potential biomarkers of response and resistance. We noted that although there were few patients with *CDKN2A* mutations, this was statistically associated with progressive disease on P+T. *CDKN2A* mutations were significantly associated with shorter PFS and OS, *KRAS* with significantly shorter OS, and *PIK3CA* and KRAS demonstrated a trend toward shorter PFS. Our findings about *CDKN2A* need to be confirmed on larger data sets, as this finding was based on only three patients. However, this finding has scientific rationale and *CDKN2A* alterations have been associated with shorter PFS with other targeted therapies such as FGFR inhibitor futibatinib.^42^ Furthermore, we had already shown in tumor co-alteration studies that *KRAS* mutations were significantly associated with decreased ORR and decreased PFS, while *PIK3CA* demonstrated a trend toward a decrease in PFS.^31^ All of these findings may have therapeutic implications for combination therapy as well.

Another major advantage of liquid biopsies is the ability to detect acquired resistance alterations that may be polyclonal. We had post-progression samples from 21 patients, noting that this time point was at 8 weeks for eight patients. Of these 21 patients, 20 had *ERBB2* amplification detectable at C1D1 ctDNA and 18 had *ERBB2* detectable at progression. Notably, four patients acquired *ERBB2* mutations previously not detected in the plasma or on tissue sampling. None of the mutations detected were at trastuzumab or pertuzumab binding sites; however, we cannot exclude that the mutations could not have allosterically interfered with binding of trastuzumab or pertuzumab. Furthermore, two patients demonstrated emergence of oncogenic *ERBB2* mutations, raising a potential role for the addition of small molecule inhibitors.

Our study also demonstrated other potential mechanisms of acquired resistance. Five patients (23.8%) developed detectable MAPK pathway alterations (*KRAS, NRAS, MAP2K1*, and *BRAF*), six (28.6%) developed PI3K pathway alterations (*PTEN, TSC1, PI3KCA*, and *MTOR*), and several others developed alterations in other receptor tyrosine kinases. Interestingly, six (28.6%) developed *MET* alterations (including four *MET* amplifications); *MET* amplification has already been reported to be associated with resistance to EGFR and HER2 inhibitors.^43–45^ Notably, three of the *MET* amplifications emerged in early progressors, suggesting selection of preexisting resistant clones. Notably, two of four patients had prior EGFR therapy; thus, these may have emerged during prior EGFR inhibitor therapy. Further study is needed to see if combinations with MET inhibitors, KRAS inhibitors, or AKT/mTOR inhibitors could be pursued to overcome acquired resistance in a personalized fashion.

Our study has multiple limitations. Our study cohort was small and represented only half of the patients treated in the HER2-positive CRC cohort of the study due to introduction of ctDNA testing later in the study. However, our patients were treated with an active regimen with responders and non-responders, allowing us to correlate ctDNA results with response outcomes. Our enrollment was based on archival tissue and thus we are unable to differentiate between genomic evolution and false negative assays. We used a small ctDNA panel but one that has been previously well characterized. Finally, only 7 patients with a PR had post-progression liquid biopsies, limiting our ability for discovery of mechanisms of acquired resistance.

Our study demonstrated that ctDNA can detect *ERBB2* amplification in ~70% of the patients with *ERBB2*-amplified advanced CRC, suggesting that while *ERBB2* amplification is detectable in most patients in the ctDNA, tissue testing is necessary to capture all *ERBB2*-amplified patients. ctDNA MR was associated with better PFS and OS. ctDNA co-alterations allowed insight into mechanisms of intrinsic and acquired resistance and may provide directions for combination therapies.

## Supplementary Material

Supplementary Figure 1

Supplementary Figure 2

Supplementary Table 1

Supplementary Table 2

Supplementary Table 3

Supplementary Table 4

## Data Availability

For eligible studies qualified researchers may request access to individual patient level clinical data through a data request platform. At the time of this writing this request platform is Vivli. https://vivli.org/ourmember/roche/. For up to date details on Roche’s Global Policy on the Sharing of Clinical Information and how to request access to related clinical study documents, see here: https://go.roche.com/data_sharing. Anonymized records for individual patients across more than one data source external to Roche cannot, and should not, be linked due to a potential increase in risk of patient re-identification.

## Translational Relevance

ctDNA may have a role in patient selection for HER2-targeted therapy, response monitoring as well assessing mechanisms of resistance to P+T.

## Data Availability

The data generated in this study are available upon request from the corresponding author.
