# Supplementary material for: Circulating Tumor DNA Analysis in ERBB2-Amplified Colorectal Cancer: Biomarker Analysis of the MyPathway Trial
Source: Clin Cancer Res. Author manuscript; Available in PMC 2025 Sep 2. (PMC7618057; doi:10.1158/1078-0432.CCR-24-2763)
Supplement: Supplementary Figure 1 [file EMS207949-supplement-Supplementary_Figure_1.pptx]

## Slide 1
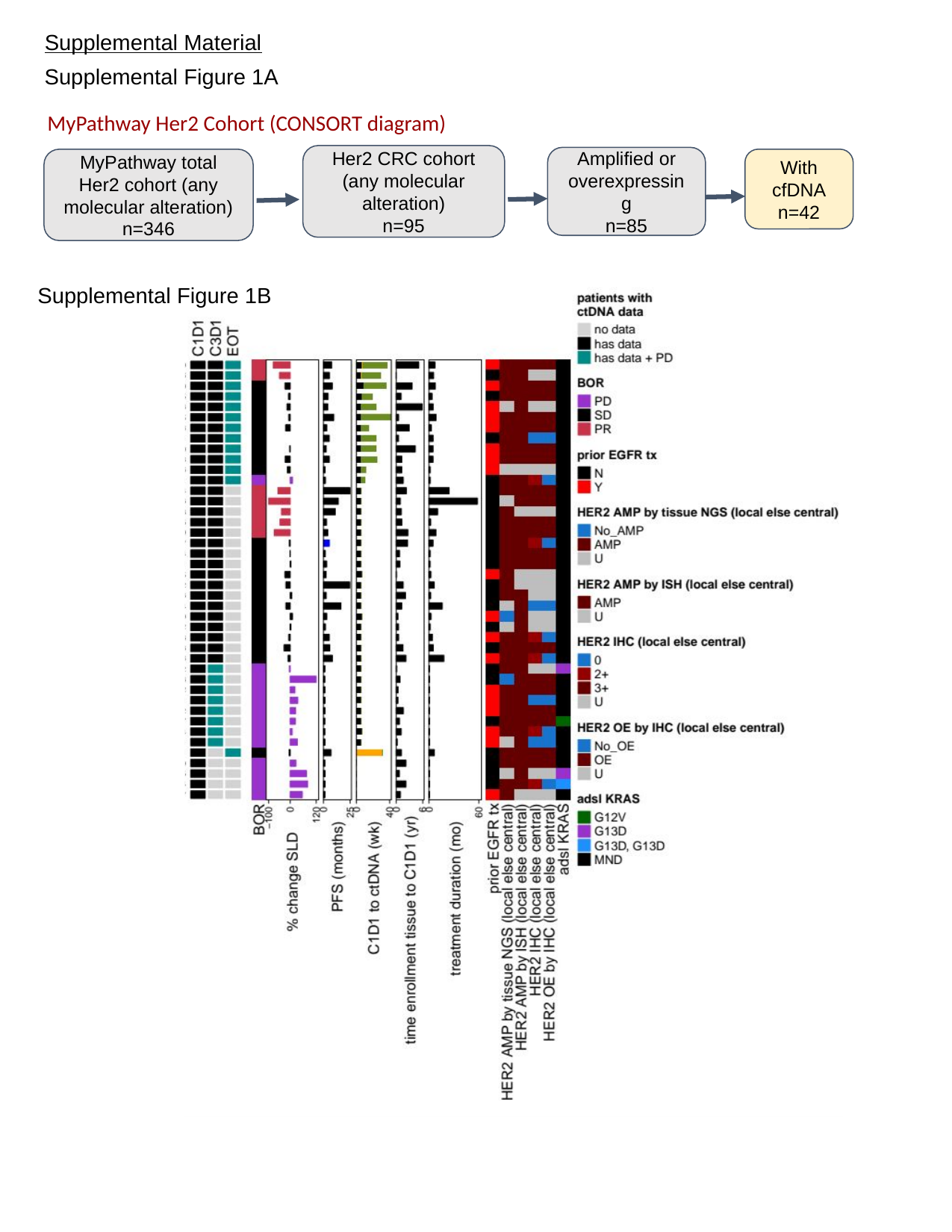

Supplemental Material
Supplemental Figure 1A
MyPathway Her2 Cohort (CONSORT diagram)
Her2 CRC cohort (any molecular alteration)
n=95
Amplified or overexpressing
n=85
MyPathway total Her2 cohort (any molecular alteration)
n=346
With cfDNA
n=42
Supplemental Figure 1B
