# Supplementary material for: Circulating Tumor DNA Analysis in ERBB2-Amplified Colorectal Cancer: Biomarker Analysis of the MyPathway Trial
Source: Clin Cancer Res. Author manuscript; Available in PMC 2025 Sep 2. (PMC7618057; doi:10.1158/1078-0432.CCR-24-2763)
Supplement: Supplementary Figure 2 [file EMS207949-supplement-Supplementary_Figure_2.pptx]

## Slide 1
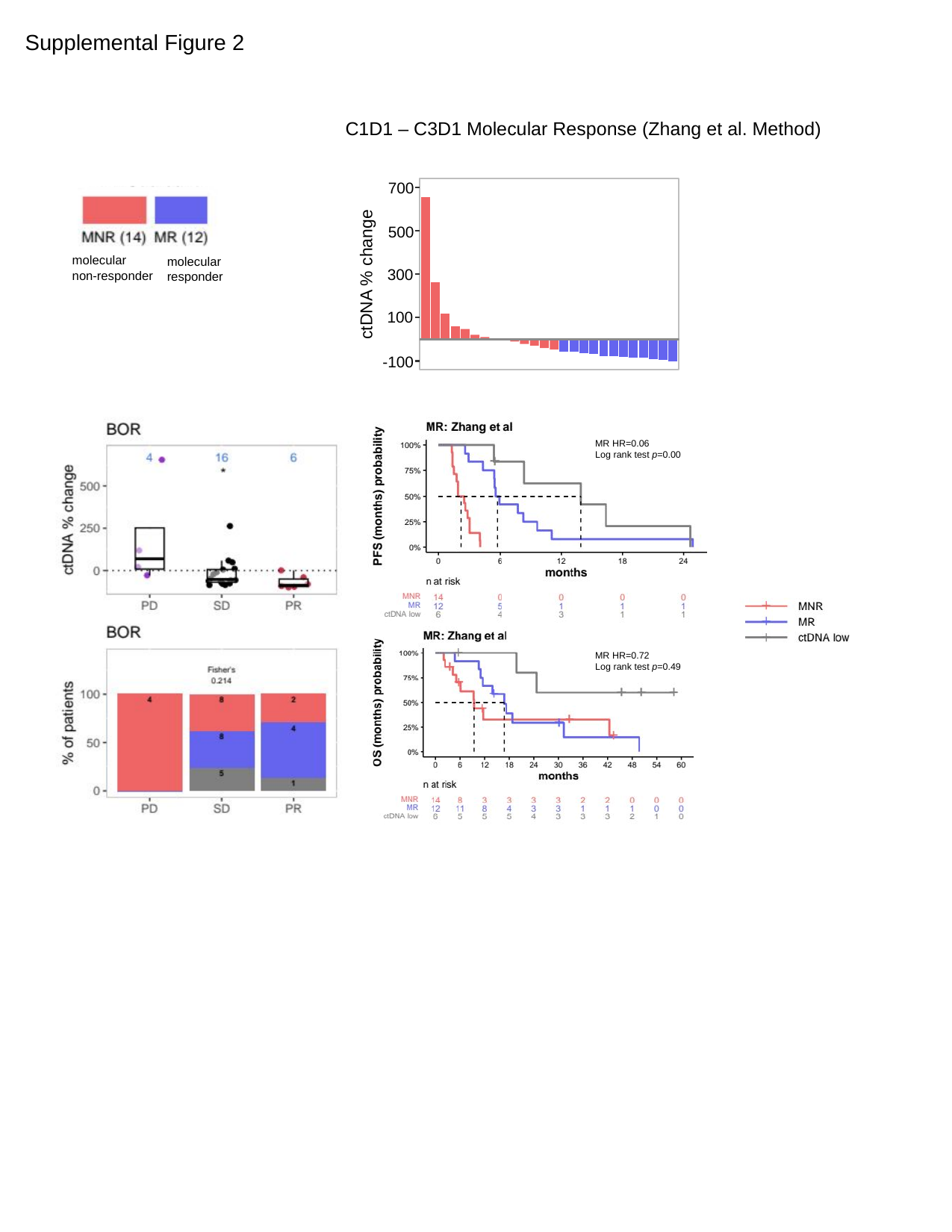

Supplemental Figure 2
C1D1 – C3D1 Molecular Response (Zhang et al. Method)
700
ctDNA % change
molecular non-responder
500
molecular responder
300
100
-100
MR HR=0.06
Log rank test p=0.00
MR HR=0.72
Log rank test p=0.49
