# Supplementary material for: Circulating Tumor DNA Analysis in ERBB2-Amplified Colorectal Cancer: Biomarker Analysis of the MyPathway Trial
Source: Clin Cancer Res. Author manuscript; Available in PMC 2025 Sep 2. (PMC7618057; doi:10.1158/1078-0432.CCR-24-2763)
Supplement: Supplementary Table 1 [file EMS207949-supplement-Supplementary_Table_1.pptx]

## Slide 1
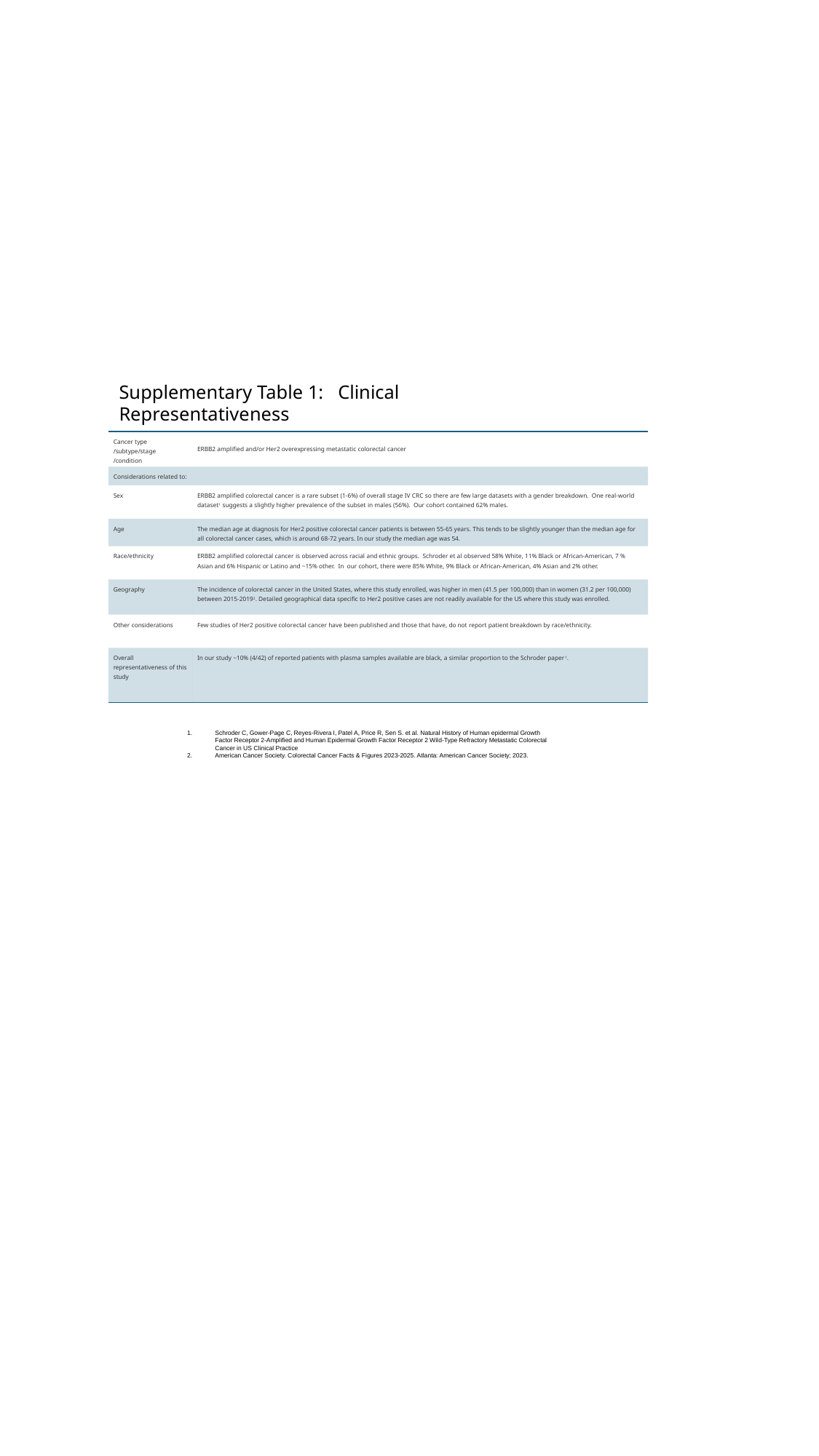

Supplementary Table 1: Clinical Representativeness
| Cancer type /subtype/stage /condition | ERBB2 amplified and/or Her2 overexpressing metastatic colorectal cancer |
| --- | --- |
| Considerations related to: | |
| Sex | ERBB2 amplified colorectal cancer is a rare subset (1-6%) of overall stage IV CRC so there are few large datasets with a gender breakdown. One real-world dataset1 suggests a slightly higher prevalence of the subset in males (56%). Our cohort contained 62% males. |
| Age | The median age at diagnosis for Her2 positive colorectal cancer patients is between 55-65 years. This tends to be slightly younger than the median age for all colorectal cancer cases, which is around 68-72 years. In our study the median age was 54. |
| Race/ethnicity | ERBB2 amplified colorectal cancer is observed across racial and ethnic groups. Schroder et al observed 58% White, 11% Black or African-American, 7 % Asian and 6% Hispanic or Latino and ~15% other. In our cohort, there were 85% White, 9% Black or African-American, 4% Asian and 2% other. |
| Geography | The incidence of colorectal cancer in the United States, where this study enrolled, was higher in men (41.5 per 100,000) than in women (31.2 per 100,000) between 2015-20192. Detailed geographical data specific to Her2 positive cases are not readily available for the US where this study was enrolled. |
| Other considerations | Few studies of Her2 positive colorectal cancer have been published and those that have, do not report patient breakdown by race/ethnicity. |
| Overall representativeness of this study | In our study ~10% (4/42) of reported patients with plasma samples available are black, a similar proportion to the Schroder paper1. |
Schroder C, Gower-Page C, Reyes-Rivera I, Patel A, Price R, Sen S. et al. Natural History of Human epidermal Growth Factor Receptor 2-Amplified and Human Epidermal Growth Factor Receptor 2 Wild-Type Refractory Metastatic Colorectal Cancer in US Clinical Practice
American Cancer Society. Colorectal Cancer Facts & Figures 2023-2025. Atlanta: American Cancer Society; 2023.
