# Supplementary figures and images for: Circulating Tumor DNA Analysis in ERBB2-Amplified Colorectal Cancer: Biomarker Analysis of the MyPathway Trial
Source: Clin Cancer Res. Author manuscript; Available in PMC 2025 Sep 2. (PMC7618057; doi:10.1158/1078-0432.CCR-24-2763)

## Slide 1
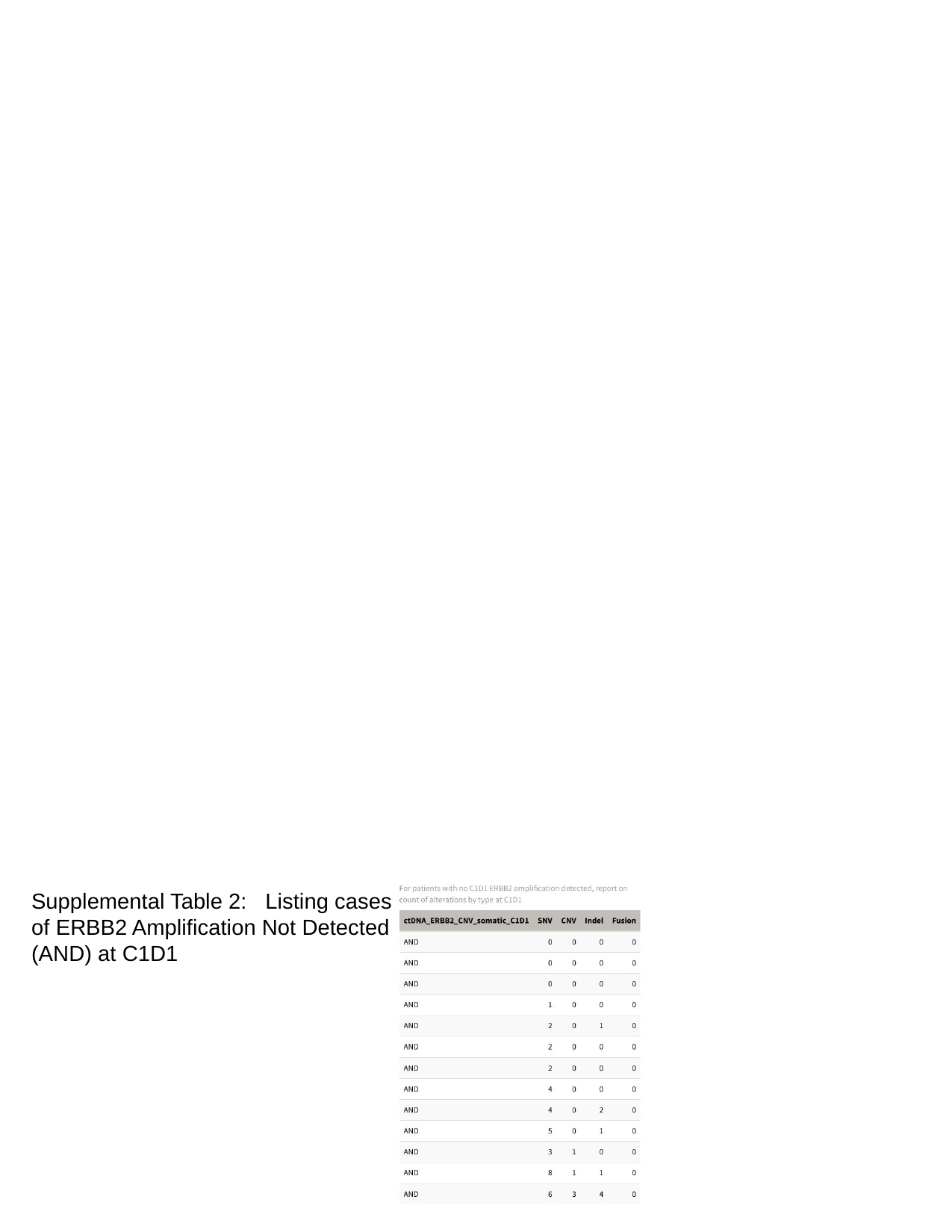

Supplemental Table 2: Listing cases of ERBB2 Amplification Not Detected (AND) at C1D1

Supplement: Supplementary Table 2 [file EMS207949-supplement-Supplementary_Table_2.pptx]
