# Supplementary material for: Circulating Tumor DNA Analysis in ERBB2-Amplified Colorectal Cancer: Biomarker Analysis of the MyPathway Trial
Source: Clin Cancer Res. Author manuscript; Available in PMC 2025 Sep 2. (PMC7618057; doi:10.1158/1078-0432.CCR-24-2763)
Supplement: Supplementary Table 3 [file EMS207949-supplement-Supplementary_Table_3.pptx]

## Slide 1
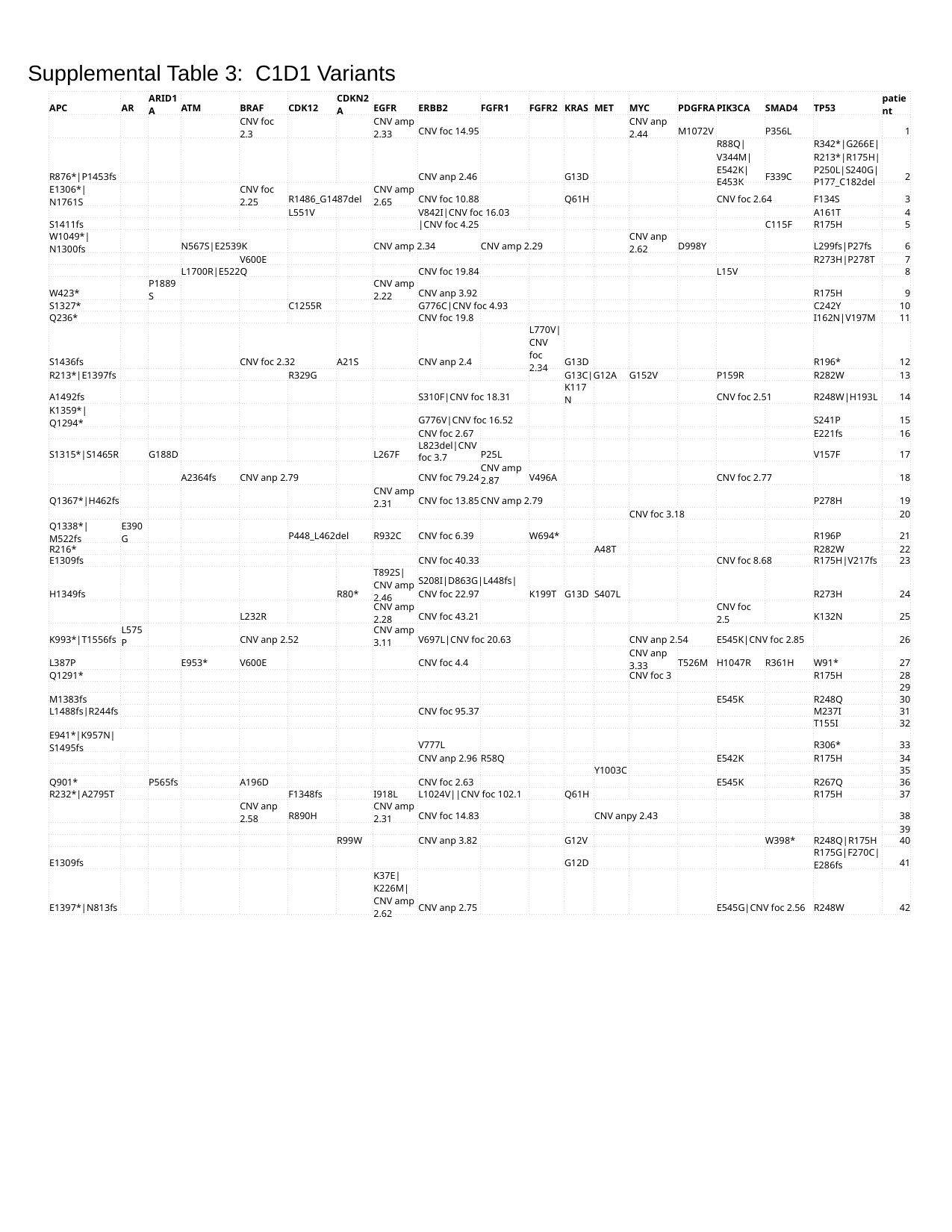

Supplemental Table 3: C1D1 Variants
| APC | AR | ARID1A | ATM | BRAF | CDK12 | CDKN2A | EGFR | ERBB2 | FGFR1 | FGFR2 | KRAS | MET | MYC | PDGFRA | PIK3CA | SMAD4 | TP53 | patient |
| --- | --- | --- | --- | --- | --- | --- | --- | --- | --- | --- | --- | --- | --- | --- | --- | --- | --- | --- |
| | | | | CNV foc 2.3 | | | CNV amp 2.33 | CNV foc 14.95 | | | | | CNV anp 2.44 | M1072V | | P356L | | 1 |
| R876\*|P1453fs | | | | | | | | CNV anp 2.46 | | | G13D | | | | R88Q|V344M|E542K|E453K | F339C | R342\*|G266E|R213\*|R175H|P250L|S240G|P177\_C182del | 2 |
| E1306\*|N1761S | | | | CNV foc 2.25 | R1486\_G1487del | | CNV amp 2.65 | CNV foc 10.88 | | | Q61H | | | | CNV foc 2.64 | | F134S | 3 |
| | | | | | L551V | | | V842I|CNV foc 16.03 | | | | | | | | | A161T | 4 |
| S1411fs | | | | | | | | |CNV foc 4.25 | | | | | | | | C115F | R175H | 5 |
| W1049\*|N1300fs | | | N567S|E2539K | | | | CNV amp 2.34 | | CNV amp 2.29 | | | | CNV anp 2.62 | D998Y | | | L299fs|P27fs | 6 |
| | | | | V600E | | | | | | | | | | | | | R273H|P278T | 7 |
| | | | L1700R|E522Q | | | | | CNV foc 19.84 | | | | | | | L15V | | | 8 |
| W423\* | | P1889S | | | | | CNV amp 2.22 | CNV anp 3.92 | | | | | | | | | R175H | 9 |
| S1327\* | | | | | C1255R | | | G776C|CNV foc 4.93 | | | | | | | | | C242Y | 10 |
| Q236\* | | | | | | | | CNV foc 19.8 | | | | | | | | | I162N|V197M | 11 |
| S1436fs | | | | CNV foc 2.32 | | A21S | | CNV anp 2.4 | | L770V|CNV foc 2.34 | G13D | | | | | | R196\* | 12 |
| R213\*|E1397fs | | | | | R329G | | | | | | G13C|G12A | | G152V | | P159R | | R282W | 13 |
| A1492fs | | | | | | | | S310F|CNV foc 18.31 | | | K117N | | | | CNV foc 2.51 | | R248W|H193L | 14 |
| K1359\*|Q1294\* | | | | | | | | G776V|CNV foc 16.52 | | | | | | | | | S241P | 15 |
| | | | | | | | | CNV foc 2.67 | | | | | | | | | E221fs | 16 |
| S1315\*|S1465R | | G188D | | | | | L267F | L823del|CNV foc 3.7 | P25L | | | | | | | | V157F | 17 |
| | | | A2364fs | CNV anp 2.79 | | | | CNV foc 79.24 | CNV amp 2.87 | V496A | | | | | CNV foc 2.77 | | | 18 |
| Q1367\*|H462fs | | | | | | | CNV amp 2.31 | CNV foc 13.85 | CNV amp 2.79 | | | | | | | | P278H | 19 |
| | | | | | | | | | | | | | CNV foc 3.18 | | | | | 20 |
| Q1338\*|M522fs | E390G | | | | P448\_L462del | | R932C | CNV foc 6.39 | | W694\* | | | | | | | R196P | 21 |
| R216\* | | | | | | | | | | | | A48T | | | | | R282W | 22 |
| E1309fs | | | | | | | | CNV foc 40.33 | | | | | | | CNV foc 8.68 | | R175H|V217fs | 23 |
| H1349fs | | | | | | R80\* | T892S|CNV amp 2.46 | S208I|D863G|L448fs|CNV foc 22.97 | | K199T | G13D | S407L | | | | | R273H | 24 |
| | | | | L232R | | | CNV amp 2.28 | CNV foc 43.21 | | | | | | | CNV foc 2.5 | | K132N | 25 |
| K993\*|T1556fs | L575P | | | CNV anp 2.52 | | | CNV amp 3.11 | V697L|CNV foc 20.63 | | | | | CNV anp 2.54 | | E545K|CNV foc 2.85 | | | 26 |
| L387P | | | E953\* | V600E | | | | CNV foc 4.4 | | | | | CNV anp 3.33 | T526M | H1047R | R361H | W91\* | 27 |
| Q1291\* | | | | | | | | | | | | | CNV foc 3 | | | | R175H | 28 |
| | | | | | | | | | | | | | | | | | | 29 |
| M1383fs | | | | | | | | | | | | | | | E545K | | R248Q | 30 |
| L1488fs|R244fs | | | | | | | | CNV foc 95.37 | | | | | | | | | M237I | 31 |
| | | | | | | | | | | | | | | | | | T155I | 32 |
| E941\*|K957N|S1495fs | | | | | | | | V777L | | | | | | | | | R306\* | 33 |
| | | | | | | | | CNV anp 2.96 | R58Q | | | | | | E542K | | R175H | 34 |
| | | | | | | | | | | | | Y1003C | | | | | | 35 |
| Q901\* | | P565fs | | A196D | | | | CNV foc 2.63 | | | | | | | E545K | | R267Q | 36 |
| R232\*|A2795T | | | | | F1348fs | | I918L | L1024V||CNV foc 102.1 | | | Q61H | | | | | | R175H | 37 |
| | | | | CNV anp 2.58 | R890H | | CNV amp 2.31 | CNV foc 14.83 | | | | CNV anpy 2.43 | | | | | | 38 |
| | | | | | | | | | | | | | | | | | | 39 |
| | | | | | | R99W | | CNV anp 3.82 | | | G12V | | | | | W398\* | R248Q|R175H | 40 |
| E1309fs | | | | | | | | | | | G12D | | | | | | R175G|F270C|E286fs | 41 |
| E1397\*|N813fs | | | | | | | K37E|K226M|CNV amp 2.62 | CNV anp 2.75 | | | | | | | E545G|CNV foc 2.56 | | R248W | 42 |
