# Supplementary material for: Circulating Tumor DNA Analysis in ERBB2-Amplified Colorectal Cancer: Biomarker Analysis of the MyPathway Trial
Source: Clin Cancer Res. Author manuscript; Available in PMC 2025 Sep 2. (PMC7618057; doi:10.1158/1078-0432.CCR-24-2763)
Supplement: Supplementary Table 4 [file EMS207949-supplement-Supplementary_Table_4.pptx]

## Slide 1
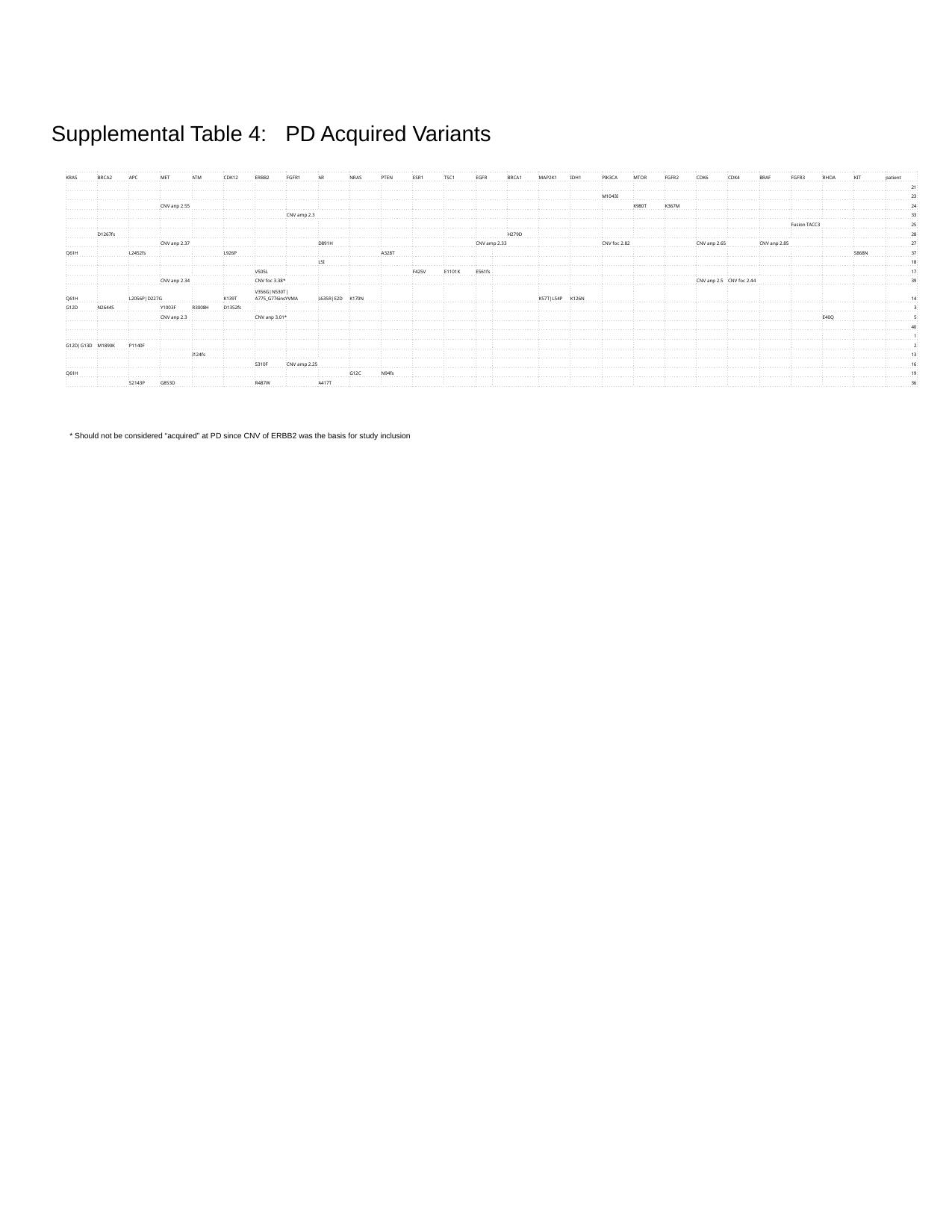

Supplemental Table 4: PD Acquired Variants
| KRAS | BRCA2 | APC | MET | ATM | CDK12 | ERBB2 | FGFR1 | AR | NRAS | PTEN | ESR1 | TSC1 | EGFR | | BRCA1 | MAP2K1 | IDH1 | PIK3CA | MTOR | FGFR2 | CDK6 | CDK4 | BRAF | FGFR3 | RHOA | KIT | patient |
| --- | --- | --- | --- | --- | --- | --- | --- | --- | --- | --- | --- | --- | --- | --- | --- | --- | --- | --- | --- | --- | --- | --- | --- | --- | --- | --- | --- |
| | | | | | | | | | | | | | | | | | | | | | | | | | | | 21 |
| | | | | | | | | | | | | | | | | | | M1043I | | | | | | | | | 23 |
| | | | CNV anp 2.55 | | | | | | | | | | | | | | | | K980T | K367M | | | | | | | 24 |
| | | | | | | | CNV amp 2.3 | | | | | | | | | | | | | | | | | | | | 33 |
| | | | | | | | | | | | | | | | | | | | | | | | | Fusion TACC3 | | | 25 |
| | D1267fs | | | | | | | | | | | | | | H279D | | | | | | | | | | | | 28 |
| | | | CNV anp 2.37 | | | | | D891H | | | | | CNV amp 2.33 | | | | | CNV foc 2.82 | | | CNV anp 2.65 | | CNV anp 2.85 | | | | 27 |
| Q61H | | L2452fs | | | L926P | | | | | A328T | | | | | | | | | | | | | | | | S868N | 37 |
| | | | | | | | | L5I | | | | | | | | | | | | | | | | | | | 18 |
| | | | | | | V505L | | | | | F425V | E1101K | E561fs | | | | | | | | | | | | | | 17 |
| | | | CNV anp 2.34 | | | CNV foc 3.38\* | | | | | | | | | | | | | | | CNV anp 2.5 | CNV foc 2.44 | | | | | 39 |
| Q61H | | L2056P|D227G | | | K139T | V356G|N530T|A775\_G776insYVMA | | L635R|E2D | K170N | | | | | | | K57T|L54P | K126N | | | | | | | | | | 14 |
| G12D | N2644S | | Y1003F | R3008H | D1352fs | | | | | | | | | | | | | | | | | | | | | | 3 |
| | | | CNV anp 2.3 | | | CNV anp 3.01\* | | | | | | | | | | | | | | | | | | | E40Q | | 5 |
| | | | | | | | | | | | | | | | | | | | | | | | | | | | 40 |
| | | | | | | | | | | | | | | | | | | | | | | | | | | | 1 |
| G12D|G13D | M1890K | P1140F | | | | | | | | | | | | | | | | | | | | | | | | | 2 |
| | | | | I124fs | | | | | | | | | | | | | | | | | | | | | | | 13 |
| | | | | | | S310F | CNV amp 2.25 | | | | | | | | | | | | | | | | | | | | 16 |
| Q61H | | | | | | | | | G12C | N94fs | | | | | | | | | | | | | | | | | 19 |
| | | S2143P | G853D | | | R487W | | A417T | | | | | | | | | | | | | | | | | | | 36 |
* Should not be considered “acquired” at PD since CNV of ERBB2 was the basis for study inclusion
